# Impact of partial cement dust replacement in unsaturated polyester: assessing material performance and waste valorization for sustainable management

**DOI:** 10.1038/s41598-025-32700-9

**Published:** 2026-01-06

**Authors:** Eslam Syala, Wagih A. Sadik, Abdel-Ghaffar M. El-Demerdash, Waffa Mekhamer, M. Essam El-Rafey

**Affiliations:** Department of Materials Science, Institute of Graduate Studies and Researches (IGSR), Alexandria University, 163 Horreya Avenue, Shatby, 21526 Alexandria Egypt

**Keywords:** Unsaturated polyester, Cement kiln dust, Composite, Mechanical properties, Water absorption, Engineering, Materials science

## Abstract

This study is a continuation of the earlier studies (The effective treatment of dye‑containing simulated wastewater by using the cement kiln dust as an industrial waste adsorbent) and (The effective remediation of heavy metal-laden wastewater by employing cement dust derived from industrial activities as a sorbent) as an attempt to provide a comprehensive image of the possible useful uses of cement dust.  In this research, a polymer composite consisting of an unsaturated polyester (UP) thermoset matrix and micro-sized cement kiln dust (CKD) as filler with an addition percentage range of 0–10% was synthesized to achieve maximum utilization of this harmful waste that influences the environment and public health. Studying the structure revealed the presence of characteristic UP and CKD peaks in all the XRD and IR spectra, confirming the physical interaction between the filler and the matrix. The inclusion of CKD up to 10% decreased the degradation temperature of the UP resin. The water absorption data for the UP–CKD composites revealed a maximum water uptake of 0.55% with nonlinear behavior compared to UP doped with other inorganic fillers. An increase in the CKD content to 10% increased the limiting oxygen index (LOI) of UP above the oxygen percentage in the air (21%), promoting the fire resistance properties of the system. Prime mechanical properties in terms of ultimate tensile strength, Young’s modulus, bending (flexural) strength, and flexural modulus decreased from 27.54 to 7.57 MPa, and from 1118.4 to 260.45 MPa, and from 25 to 11.49 MPa, and from 4.68 to 2.15 MPa, respectively, while both elongation (%) at break and hardness revealed fluctuating behavior with increasing CKD content. Poor dispersion and agglomeration, and hence low adhesion and poor bonding between the CKD filler and the UP, were the main reasons for this observed declining behavior, as exhibited from SEM illustrations. The as-prepared UP–CKD can be used in various applications where there is no need for distinctive mechanical performance, such as tables and benches manufacturing.

## Introduction

The manufacturing of Portland cement is one of the widespread industrial activities worldwide. It is considered one of the ancient industries in Egypt, where the first factory was built about 100 years ago^1^. Unfortunately, this industrial process emits various pollutants, posing significant environmental hazards to the surrounding environment. For example, producing only one ton of cement releases 0.689 tons of carbon dioxide gas and some other harmful gases like sulfur and nitrogen oxides, besides 0.07 tons of dust that is emitted to the environment^2,3^. By-pass cement kiln dust (CKD) is among the solid pollutants that are generated during the manufacturing of cement in the rotary kiln during the pyro-processing (i.e., dry process) of the ordinary Portland cement clinker. The generated percentage of CKD can reach 5–20% of the manufactured cement^4,5^. It is considered special waste, as acknowledged by the agency of Environmental Protection due to its destructive effects on beings^6^. Its disposal represents a huge problem from the mass production, environmental, health, cost, and burial points of view. The movement of rain and wind in the location of the factory can exacerbate this environmental problem by increasing the possibility of dust spreading over a larger surrounding area^7^. In Egypt (as an example), the raw materials within the quarries contain high values of alkalis and sulfates, which finally result in producing large quantities of CKD. To imagine the extent of the problem, one of the Portland Cement Companies in the west of Alexandria, Egypt, for example, produces 120 tons/day of CKD. This quantity is transported daily to special dumps and buried, then covered with clay to prevent volatility or effects on underground water. However, CKD should be separated and discharged before incorporation in the clinker because of its high content of salts and chlorides^5,8^. Attempts have been made to reuse and apply CKD in various applications. These attempts were cautious attempts to avoid any possible harmful effects of CKD on the applied uses. For example, Hassan et al. intended to reuse 15% of CKD as a partial substitute in concrete, but the experiments revealed that it couldn’t increase by more than 5% by weight to avoid the corrosion of steel because of increasing the possibility of chloride permeability and decreasing the electrical resistivity of CKD/concrete. Other possible risks of increased CKD replacement in concrete include reduced compressive strength and increased water demand to reach the standard consistency, as concluded by Najim et al.^8^. Hammood et al. used CKD as a reinforcement up to 16% by weight in an Al–Mg alloy via a powder metallurgy technique. The results showed a 74% improvement in hardness and a 59% decrease in thermal conductivity, while the wear rate, friction coefficient, and porosity lowered by 78%, 74%, and 50%, successively^9^. On the level of polymeric composites, Khozemy et al. studied the effect of CKD incorporation on the different properties of polyethylene composites. They attained a maximum value of tensile strength of only 10.7 MPa with the addition of CKD up to 30 phr^10^. Elhrari et al. examined the CKD addition influence on the mechanical properties of high-density polyethylene composites. They observed that the tensile strength, elastic modulus, and impact strength of the composites decreased with further increase of CKD filler percentage^11^. These findings underscore the potential of CKD as a filler in polymer composites; however, its viability as a sustainable filler also depends on understanding its generation method through dominant manufacturing processes. The hydro process (i.e., wet process) was preferred in the past over the dry process, but on the other hand, it is more costly due to the need for more energy (40% more energy compared to the dry process) for drying the feed materials before clinker burning^12^. For that reason, the dry process is dominant in the cement industry due to its energy efficiency, lower overall production costs, and environmental benefits^13^. These manufacturing advantages position CKD-generated filler as an ideal component within engineered polymer composites, which involve the strategic design and production of distinct matrix and reinforcing phases on an industrial scale. Composites are materials consisting of two or more distinct phases at a macroscopic level: a continuous matrix phase and a dispersed reinforcing phase^14^. Where composites are completely engineered products, they start with simulation, design, and then processing through manufacturing planning and execution on an industry scale. This sequence helps in the efficient prediction of the material’s properties and behavior with a high level of accuracy. There is a need to develop the composites creation and industry where they are used nearly everywhere nowadays. The composites industry provides industrialization support and assets for companies in all sectors, enabling them to develop new solutions for their clients’ problems, at a time when there is a gap between available resources and the company’s needs. Taking into account both cost and applications, unsaturated polyester (UP) is characterized by its good mechanical properties, chemical resistance, versatility, and ease of processing, making it a popular choice for various applications, compared to versatile thermoplastics (like Polypropylene, polyethylene) that are generally expensive resins. Therefore, creating a composite system (using the UP as a matrix) that utilizes the CKD as a filler has the advantage over other filler materials due to 1) achieving environmental sustainability, and 2) cost-effectiveness (based on the CKD availability compared to other conventional fillers that often require extensive purification, treatment, or synthesis^15^). Consequently, the study advances beyond these previous researches by simultaneously addressing three key challenges: (1) the development of an environmentally benign processing route applying cold mixing approach that eliminates harmful volatile organic compounds (VOC) emissions typical of thermoset composite fabrication; (2) the direct utilization of untreated CKD without chemical or thermal preprocessing, thereby reducing the production costs and simplifying manufacturing logistics; and (3) the comprehensive characterization of micro-scale, non-fibrous CKD filler interactions within the unsaturated polyester matrix, with explicit quantification of the fire-resistance/mechanical-property trade-off. This integrated approach yielded valuable insights into filler-matrix adhesion mechanisms at the micro-scale while establishing sustainable processing conditions applicable to large-scale cement kiln dust valorization.

The study also aligned with the Sustainable Development Goals (SDGs), particularly the health and environmental goals.

## Experimental section (Materials, methods, and characterization)

### The starting materials

**Fig. 1:**
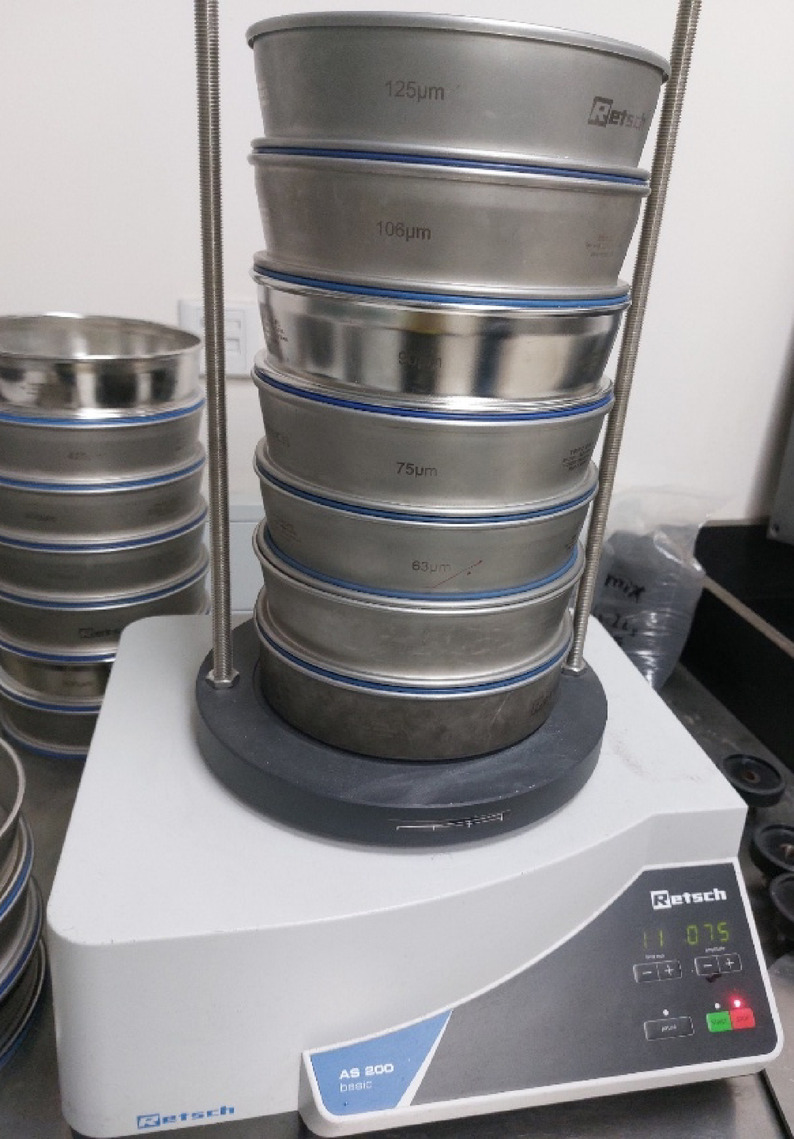
The applied Vibratory Sieve Shaker, RETSCH.

The used matrix was a slightly yellow general-purpose unsaturated polyester (UP) resin, T-Poly 620, with a medium viscosity (400–600 cP), a gelling time of 10–20 min, and medium reactivity. Cobalt Octoate (6%), as an accelerator, was well mixed into the resin. Finally, a general-purpose colorless solution of MOLPEROX F50 Methyl Ethyl Ketone Peroxide (MEKP) in Dimethyl Phthalate with a density of 1.18 ± 0,005 g/cm^3^, 24 cP viscosity, and Peroxide Content of 30–37% was used for curing UP matrix as a catalyst to start the chain reaction and initiate the crosslinking between both the polyester and styrene particles. UP, Cobalt, as well as MEKP, were imported from TBP Chemicals, Turkey. CKD, which was used as a filler, was bought from the Alexandria Portland Cement Company (TITAN), west of Alexandria province, Egypt. Its chemical formulation was attained by an Axios Max Panalytical wavelength-dispersive X-ray fluorescence spectrometer, Malvern Panalytical, Netherlands, and it is presented in Table 1. The CKD powder was also sorted related to a particle size point of view by using a Vibratory Sieve Shaker, AS 200 basic, RETSCH, Germany, as depicted in Fig. 1. The sorting was performed using 125, 106, 90, 75, 63, and 53 μm sieves. The results revealed that about 80% of the powder was retained on a 75 μm sieve, which is in line with the literature that also acknowledged this particle size distribution for the CKD^16^. About 5% was retained on a 125 μm sieve, 2% was retained on a 106 μm sieve, 4% was retained on a 90 μm sieve, 3% was retained on a 63 μm sieve, and the rest of the CKD powder (≈ 6%) was retained on a 53 μm sieve.

**Table 1:** Chemical composition of cement kiln dust as obtained by XRF.

Oxide	(%)
SiO_2_	14.59
Al_2_O_3_	2.99
Fe_2_O_3_	3.60
CaO	48.24
MgO	3.32
SO_3_	4.42
Na_2_O	2.59
K_2_O	6.55
Cl	8.58
LOI	7.58

### Methods (preparation of samples)

**Fig. 2:**
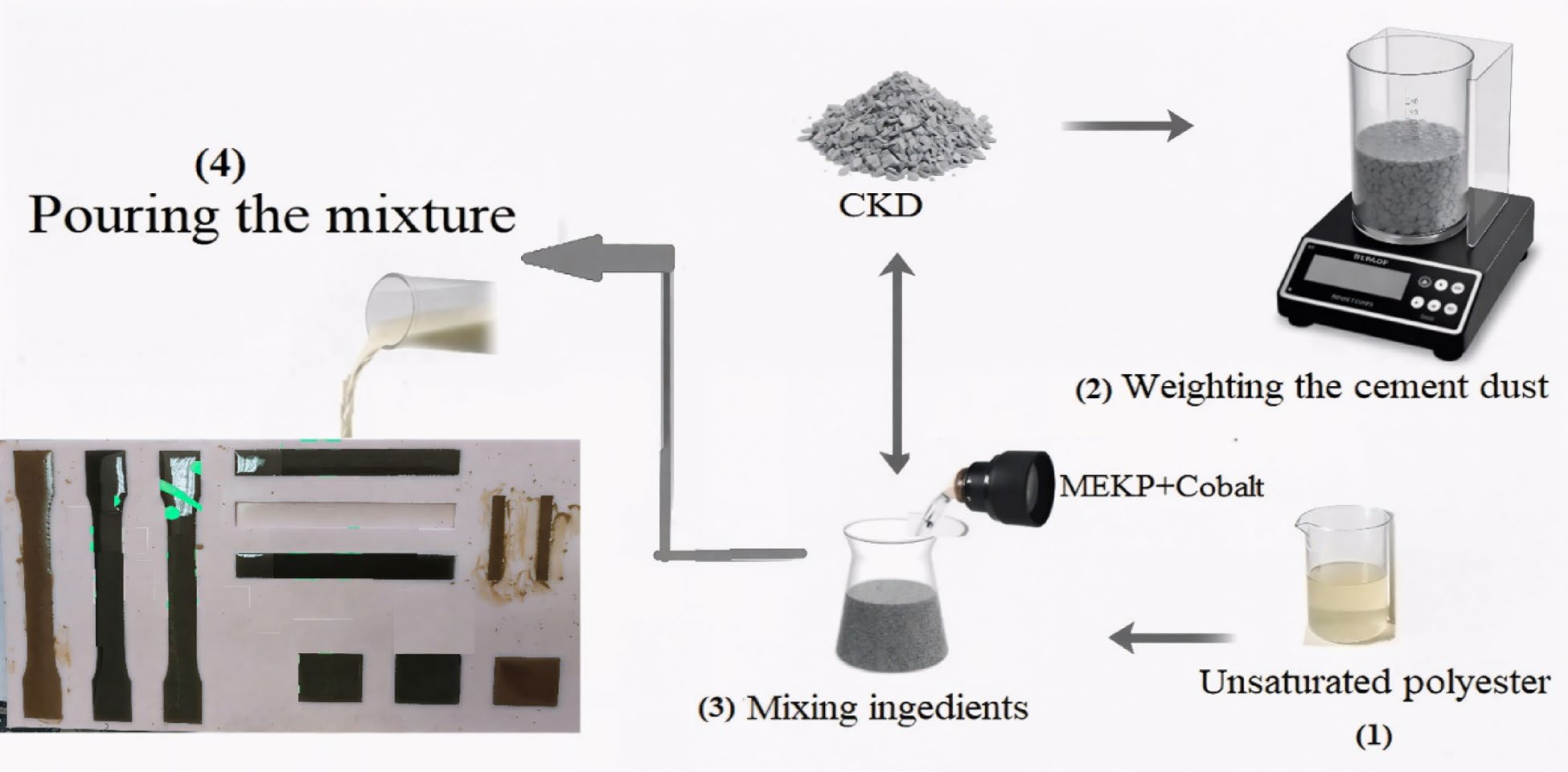
The preparation sequence of the composites under study.

First of all, local silicon rubber with medium viscosity was used to shape the molds that were employed in pouring the UP/CKD composites as per the procedures in^17^. The samples were prepared using the conventional manual hand-lay-up method. At the earlier stage, the UP was mixed with Cobalt (Akzo) for about five minutes, obtaining a homogeneous mix, then the predetermined filler amount was weighed and added gradually with continuous mixing until relative complete uptake. Finally, MEKP solution was added and mixed for about one to two minutes to catalyze the reaction, harden the batch, and obtain the final composites that were prepared according to the designation in Table 2. When the percentage of CKD increased above 10%, as was performed during the experimentation, there were problems related to the composite (solidification-appearance, etc.); thus, this proportion of filler (i.e., 10%) was the upper limit to keep an acceptable composition for the samples of the composite. This might be due to the inhibition of the curing reaction of UP by CKD. So, increasing the amounts of both the Cobalt accelerator and the MEKP hardener helped in compensating for this inhibition. Besides, higher concentrations of CKD resulted in a larger surface area, which can absorb more of the curing agents, so increasing the amounts of accelerator and hardener was mandatory to provide enough conditions for the reaction. The mixtures were poured into various shapes according to the requirements of the testing conditions and characterization standards and left to solidify in ambient air. The preparation sequence was displayed in Fig. 2, portraying each step with photos following the procedures in the previous works^18–21^.

**Table 2:** The composition of the prepared UP–CKD composites.

Sample code	Participating percentages (%)			
	UP matrix	CKD filler	Cobalt accelerator	MEKP hardener
UP–CKD 0%	100	0	0.5	2
UP–CKD 2%	98	2	0.5	2
UP–CKD 4%	96	4	0.5	2
UP–CKD 6%	94	6	0.5	2
UP–CKD 8%	92	8	0.75	3
UP–CKD 10%	90	10	1	4

### Characterization

#### X-Ray Diffraction (XRD)

The microstructure of both the net UP as well as the composites was examined by the XRD analysis using a D8 Discover, Bruker advanced X-ray solutions device, Germany, with Cu Kα radiation (wavelength λ = 0.15406 nm), and a nickel filter at 40 kV and 40 mA within the 2θ scanning range from 10 to 90° and a 0.02° (2θ) step size.

#### FTIR spectra

Fourier transform infrared (FT-IR) spectra for the cement dust powder/UP matrix/ UP–CKD various composites were obtained using a Nicolet 380 FT-IR spectrometer, USA, to investigate the interaction nature between UP and CKD. The measuring was carried out by using powder from each sample and blending it with KBr, that then pressed to shape pellets. The examination was accomplished at room temperature within a 4000–400 cm^−1^ measuring range with a precision of ± 1 cm^−1^.

#### TGA of the composite

Thermogravimetric (TGA) analysis was conducted to detect the thermal behavior of the UP–CKD composites as a function of the variations in the weight loss of the samples with temperature. The property was explored using a Universal general-purpose TA instrument Q50 device, USA. A few milligrams of samples (in the form of powder) were put in the platinum pan and heated at 10 °C/min from room temperature to 850 °C in a nitrogen atmosphere with a flow rate of 15 Psi. The isothermal temperature accuracy of the apparatus was ± 1 °C.

#### Limiting oxygen index (LOI)

The ASTM D2863-97 standard approach was performed for estimating LOI values of UP–CKD composites. This technique provides the limiting amount of oxygen (volume/volume% of a nitrogen–oxygen mixture) needed to maintain and support combustion after ignition. The index of limiting oxygen of a substance was determined by vertically putting samples of various prepared composites in a thermally resistant glass tube and analyzing their behavior towards various concentrations of oxygen. LOI is expressed in percentage as in Eq. (1).

1 $$({\text{LOI}}) = \frac{{\left[ {{\text{O}}_{2} } \right]}}{{\left( {[{\text{O}}_{2} ] + [{\text{N}}_{2} ]} \right)}} \times 100 (\% )$$

where [O₂] and [N₂] are the volumetric concentrations.

#### The density determination

The experimental densities of the as-prepared composites were determined by a simple.

Archimedes’ principle using distilled water as a buoyant liquid at room temperature and a digital balance with an accuracy of ± 0.0001, applying Eq. (2)

2 $${\text{Density }}\left( \rho \right) \, = \frac{{\text{m}}}{{{\text{V}}_{{2}} {\text{ - V}}_{{1}} }}$$

where *m* is the mass of the investigated composite in air, ${\text{V}}_{2}$ and ${\text{V}}_{1}$ are the final and initial volumes of the water, respectively, and this difference represents the displacement in the volume by the tested sample.

#### Water absorption (%)

This property refers to the absorbed quantity of water (i.e., physical or chemical reaction with water) by the sample when it is immersed in water for a definite time period under specific conditions. The test indicates the interior structure of the material. The examination was performed in line with ASTM D570-98, and the ratio of absorbed water was compute’d using the weight difference according to Eq. (3).

3 $${\text{Water}}\;{\text{absorption }}\left( \% \right) \, = \frac{{{\text{w}}_{{\text{f}}} - {\text{w}}_{{\text{i}}} }}{{{\text{w}}_{{\text{i}}} }} \times 100$$

where (${\text{w}}_{\text{i}}$) is the basic dry weight of the sample, while (${\text{w}}_{\text{f}}$) is the terminal weight after the soaking time. The test was performed on three samples for each concentration to validate the results.

#### Studying the morphology

The morphological features of the prepared blends, as well as of the pure UP, were examined by the SEM approach. The JCM-6000 Plus versatile scanning electron microscope, Japan, was used for capturing the images with magnifications of 20, 50, and 500.

### Mechanical tests

#### Tensile and bending properties testing

The tensile test provides the behavior of the UP–CKD composites when a tensile force is applied along their longitudinal vertical axis. The bending (flexure) test is a standardized technique used to verify the material’s ductility and evaluate its bending strength without fracturing. Both the tensile and the three-point bending properties for all samples were tested using a 100kN Instron 3382 dual-column floor model universal testing machine, USA, at room temperature with an accuracy of ± 0.5% of the indicated load at a crosshead rate of 0.1 MPa, where the unit is used for heavy-duty quality control testing. The accuracy of speed was ± 0.2% of the set speed (2 mm/min for the bending and 5 mm/min for the tensile testing), with the applicability of the machine for various standards such as ASTM, ISO, etc. Standardized dumbbell-shaped samples, type I dimensions of the applied standard, were used for the tensile strength test. In contrast, rectangular-shaped 95 × 15 × 5 mm specimens were used for the bending testing following the standards ASTM D638-22 and ASTM D790-03, respectively, for tensile and bending properties tests. For both tests, the measurements were repeated three times to provide more reliability and accuracy. Accordingly, Young’s modulus (E), the elongation% at the break, and bending (flexural) strength ($\sigma_{B}$) as well as the flexural modulus ($\sigma_{M}$) were determined by applying Eqs. (4@@–7) ^22–25^, respectively.

4 $${\text{E}} = \frac{\delta }{\varepsilon }$$

5 $${\text{elongation\% = }}\frac{{\Delta {\text{L}}}}{{{\text{L}}_{{\text{o}}} }}$$

6 $${\upsigma }_{{\text{B}}} { = }\frac{{{\text{3FL}}}}{{{\text{2bh}}^{{2}} }}$$

7 $${\upsigma }_{{\text{M}}} { = }\frac{{{\text{F}}L^{3} }}{{{\text{4bh}}^{{3}} }}$$

where E is Young’s modulus in (MPa), $\delta$ is the ultimate tensile strength of the sample in (MPa), and $\varepsilon$ is the strain (dimensionless), $\Delta {\text{L}}$ is the maximum displacement (change of the length) of the sample in (mm), while ${\text{L}}_{\text{o}}$ is the original length of the sample in (mm), ${\sigma }_{B}$ is the bending strength in (MPa), $\sigma_{M}$ is the flexural modulus (MPa), ${\text{F}}$ is the maximum sample applied load, ${\text{L}}$ is the extent between the two loading points (mm), ${\text{b}}$ is the width of the sample (mm), and ${\text{h}}$ is the thickness of the sample (mm).

#### Hardness measuring

The offered material’s capability to resist localized scratching or plastic deformation by indentation at a specific loading location for the composite under study was measured. A Presto hardness tester, India, was used at room temperature to obtain the (A) Shore hardness of the prepared composite samples, with dimensions 28 × 28 × 7 mm, in line with ASTM D2240. The measurement was repeated three times per sample of each concentration (which actually has three samples), that means nine times/sample, to avoid any expected error, and the average value was taken. The values that were obtained from the measurement are arbitrary, as there are no absolute standards for the hardness^26^. ANOVA statistical analysis was employed to test the statistical robustness of the data for all the blend samples.

## Results and discussion

### The XRD crystallinity analysis

**Fig. 3:**
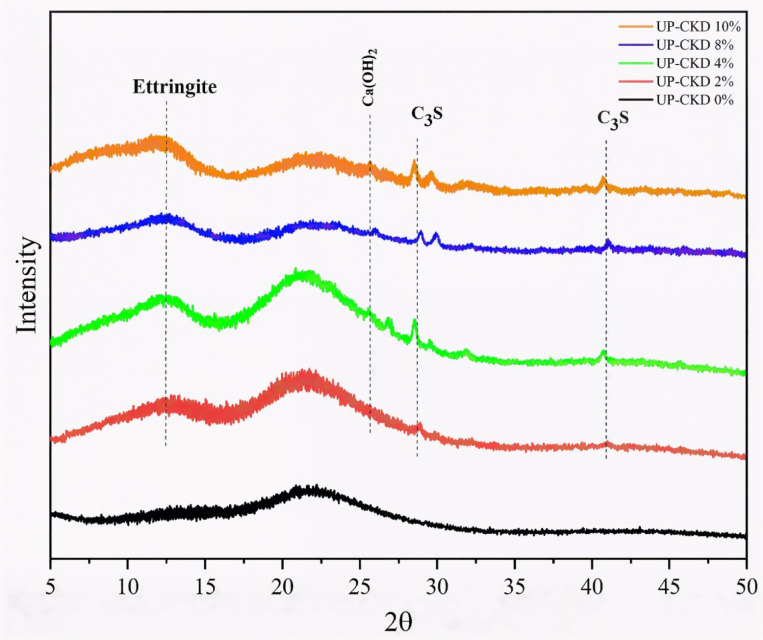
The XRD crystallography of the UP and UP–CKD composites.

Figure 3 shows the XRD crystallography of the UP and UP–CKD (2–10%) composites. The spectra reveal the amorphous nature of the neat UP, with a distinct hump-like peak at ∼ 20°^27^. The mineral phases of CKD, such as Ettringite (Ca_6_Al_2_(SO_4_)3(OH)_12_.26H_2_O) and calcium hydroxide (Ca(OH)_2_) or tricalcium silicate, appear starting from UP–CKD 2%, which represents the start of CKD filler inclusion. Peaks of other mineral phases related to CKD overlapped with UP peaks like those in the 17.5–23° range^28^. The XRD pattern affirms the physical incorporation of CKD in the UP matrix.

### FTIR results

**Fig. 4:**
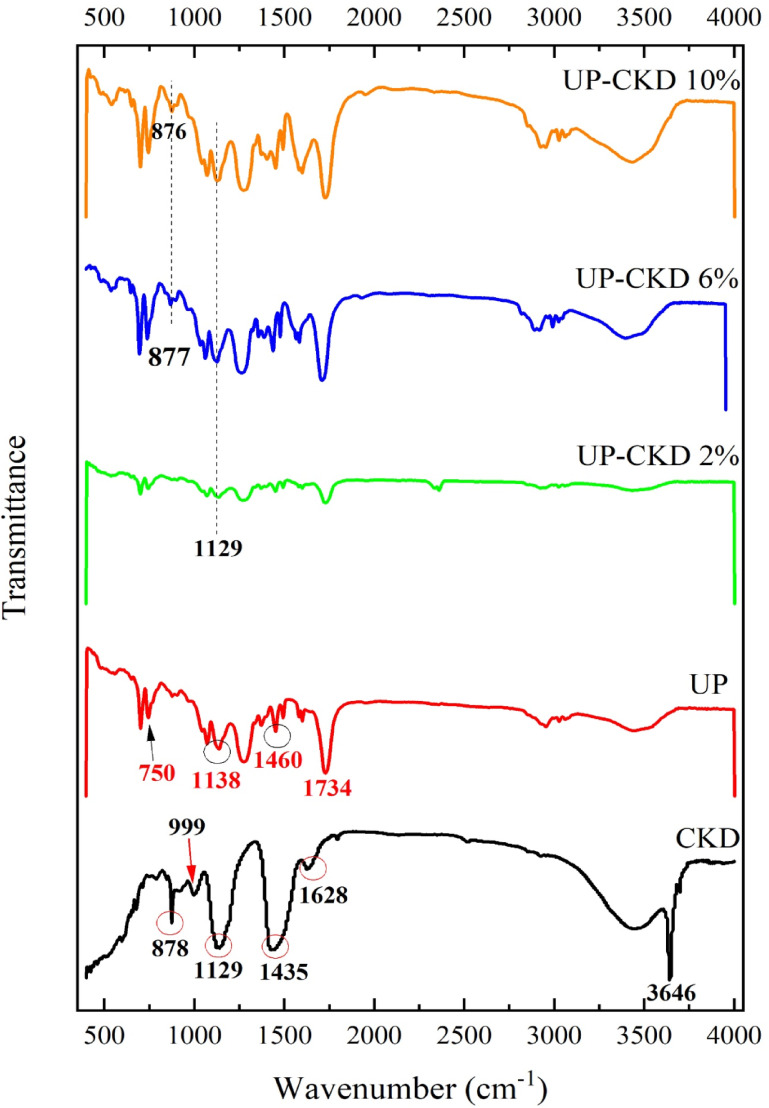
IR spectra of the CKD/UP/CKD-UP composites.

This test was executed to specify the structural units contained within the UP matrix, CKD, as well as their composites, as can be revealed in Fig. 4. Related to the CKD spectrum, the transmittance peaks close to 878 and 842, and 1129 cm^−1^ are due to the bending-in-plane vibrations of Si–O bonds in C_2_S and C_3_S phases, successively. Also, the peak at 878 cm^−1^ may overlap with the characteristic peak of CaO. The peak at 999 cm^−1^ points to the Si–O–Si bond. The peak near 1628 cm^−1^ is associated with Si–O–Si asymmetric vibration. The peak at 3646 is assigned to the O–H stretch of calcium hydroxide mineral^16,29–31^. The band ≈ 1435 cm^−1^ is allocated to the asymmetric stretching of CO_3_^2−^ of carbonate molecules resulting from calcium hydroxide and atmospheric air reaction^16,32^. Regarding the UP matrix, the band at 745 cm^−1^ is assigned to the –C–H bending developed from the 1 and 3 positions in the benzene ring. The broad band at 1140 cm^−1^ proves the existence of –C–O–C– of ester linkage. The medium absorption band at 1461 cm^−1^ is ascribed to –C–H bending. The band at 1734 cm^−1^ confirms the presence of the –C=O ester group, and it is the characteristic peak for the polyester matrix^33,34^. From the spectra of the composites, one can observe that both 878 and 1129 cm^−1^ peaks related to CKD almost maintained their positions and appeared in their sites, with the overlapping of the distinct peak of 1138 cm^−1^ relevant to UP with the 1129 cm^−1^ peak of CKD in the composites’ spectra. The small peak at 999 cm^−1^ of CKD transformed into a small shoulder in the composite spectra. The peak at 1628 cm^−1^ related to the CKD shifted backward to ≈ 1594 cm^−1^. Also, the peak at 1734 cm^−1^ attributed to the UP maintained its position in the spectra of the various composites. The presence of the distinct peaks for both UP and CKD independently in the prepared composites’ spectra confirms the physical blending of the CKD filler into the UP matrix, as can also be confirmed from the XRD spectra analysis.

### TGA of the composite (Thermal analysis)

**Fig. 5:**
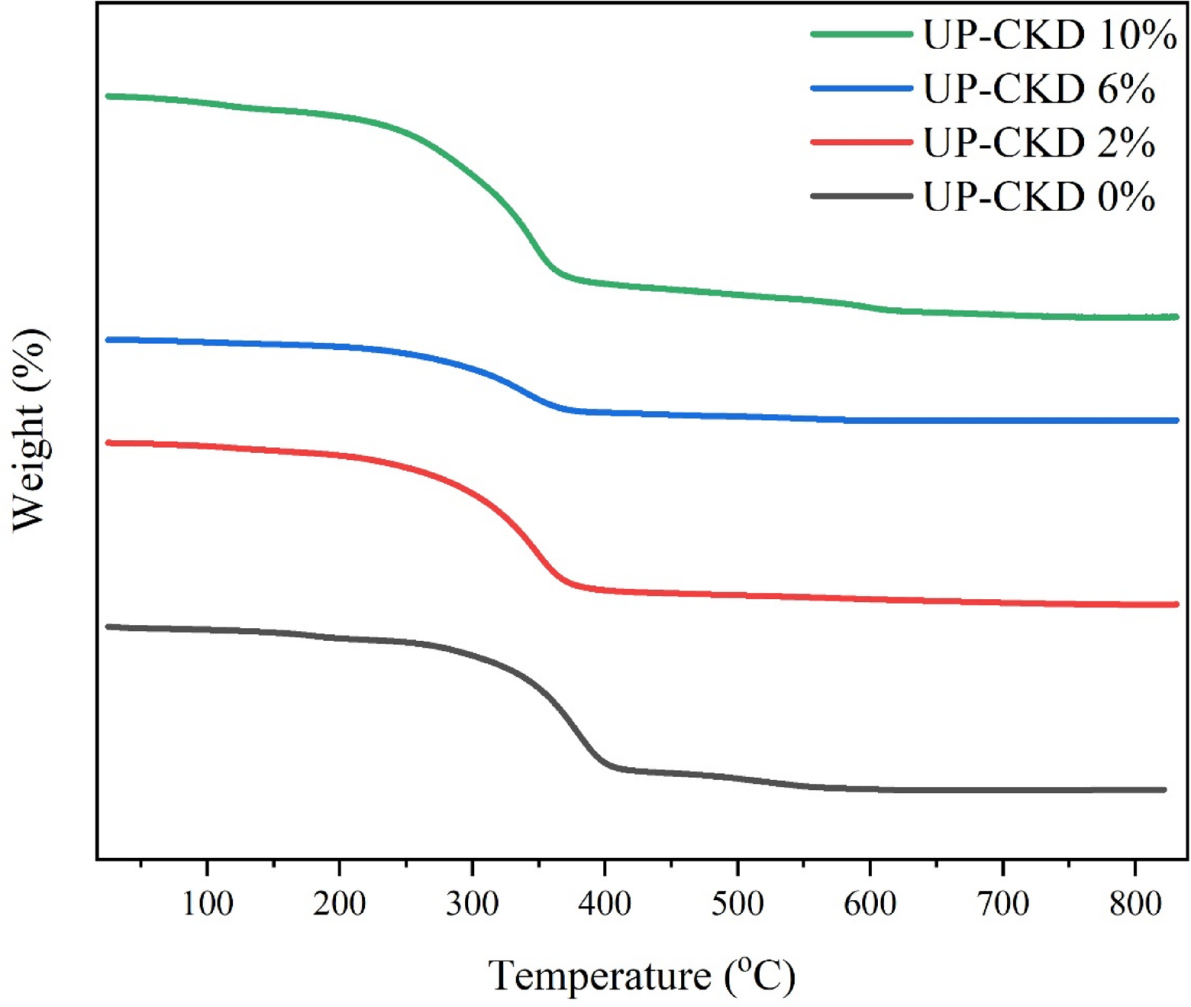
The TGA scans of the UP and UP–CKD composites.

In the present UP/CKD composites, the TGA curves (Fig. 5) depict a single major degradation step, but the onset and maximum decomposition temperatures progressively shift to lower values as CKD% increases, indicating that the polyester matrix becomes less thermally stable at higher filler loadings. To quantify the onset of degradation, the temperatures corresponding to 10% weight loss (T_d_10%) were extracted from the TGA curves for all performed compositions and recorded in Table 3. T_d_10% decreased from about 250.67 °C for neat UP to about 166.96 °C for UP‑CKD 10% composite. Where T_d_10% showed a similar downward trend, this clarifies that the incorporation of higher CKD fractions shifts the onset of thermal decomposition to lower temperatures and thus decreases the thermal stability of the polyester matrix.

**Table 3:** The physical properties of UP-CKD composites.

Sample code	Td10 (°C)	Density (g/cm^3^) ± 0.0001	Water absorption (%) ± 0.0001	LOI (%)
UP–CKD 0%	250.67	2	0.0511	19.5
UP–CKD 2%	248.42	1.80	0.1599	20
UP–CKD 4%	–	1.60	0.54739	20.5
UP–CKD 6%	216.44	1.40	0.42259	21
UP–CKD 8%	–	1.25	0.45149	21.5
UP–CKD 10%	166.96	1.08	0.19229	22

In many polymer–filler systems, well‑dispersed mineral fillers with good interfacial adhesion are reported to delay the onset of thermal degradation, promoting the thermal stability by acting as heat barriers and restricting polymer chain mobility; however, several studies also report that inorganic fillers may decrease the onset degradation temperature when they disturb the polymer network or introduce additional defects and volatile species^17,35^. This adverse effect (occurred in the present UP/CKD composites) can be attributed to a combination of mechanisms. First: CKD particles are irregular, alkaline, and relatively coarse, which promotes weak interfacial adhesion and micro‑voids initiation in the UP matrix, and such defects can facilitate heat and oxygen diffusion, accelerating chain scission during heating instead of behaving as a barrier. Secondly, CKD is hygroscopic; residual moisture and physically adsorbed water on the untreated CKD particle’s surface can volatilize at elevated temperature and catalyze early mass loss, as in the present prepared composite, especially since it was incorporated without prior drying. Thirdly, the large mismatch between the thermal expansion of UP and CKD accelerates internal thermal stresses and microcrack formation upon heating, further exposing polymer chains to degradation^36^. Fourthly, including more CKD that has a lower heat capacity (less than 730 (kJ/kg.K))^37^ to UP with high heat capacity (2296 (kJ/kg.K))^38^ interprets the observed decline in the decomposition temperature with rising CKD content, reducing the composite’s ability to buffer temperature rise and an earlier onset of decomposition. All these factors caused the composite to fail in functioning as a barrier against thermal heat and created a less stable structure under thermal stress, and diminished the blend’s overall thermal stability with rising CKD content.

### The flammability behavior

As shown in Table 3, the general trend of the prepared composites is an increase in the oxygen index with increasing CKD content in the matrix from 19.5 to 22%. This flame retardancy of the composite is attributed to the CKDs’ high silica and CaO contents (67%). Both silica and CaO are known for their role as positive flame-retardant ingredients that provide more thermal insulation and act as a diffusion barrier during the flaming process^17^. According to that, UP–CKD 10% blend revealed the most flame-retardant behavior, although it showed early breakdown in the thermal decomposition. This can be interpreted on the basis that the material degrades thermally faster but forms a protective char or residue that isolates the underlying material and limits oxygen access, making the material likely to withstand burning despite its quick decomposition when heated. Depending on this, increasing the CKD content can relatively enhance the LOI of UP by rising it above the oxygen percentage (21%) in the air^39^.

### Density measurements

Table 3 lists the values of the density of the prepared composites, which reveals a lowering trend of the values from 2 to 1.08 g/cm^3^ ± 0.0001 with the rise of the CKD filler content up to 10%. This is rational due to the replacement of a higher overall UP density matrix (1.1–1.4 g/cm^3^)^38^ with a lower bulk density CKD filler (≈ 0.60 g/cm^3^)^16^. Typically, the density of the properties determines the applications of the synthesized materials. The density range of the as-prepared composites agrees with the previously prepared UP–CKD composites^40^.

### Water absorption (%)

Table 3 shows water uptake results for the UP–CKD composites, which reveal a very low rate of water absorption capacity with non-linear behavior. The weight increase had a maximum value of 0.5%, which is a very small percentage compared to unsaturated polyester doped with cementitious materials^41^, or unsaturated polyester doped with other fillers^42,43^. Although this is a small percentage of water absorption within the material, C_3_S and C_2_S are the primary phases in cement, characterized by their hydrophilic nature, which are responsible for this water uptake^32^.

### The blend’s morphology

**Fig. 6:**
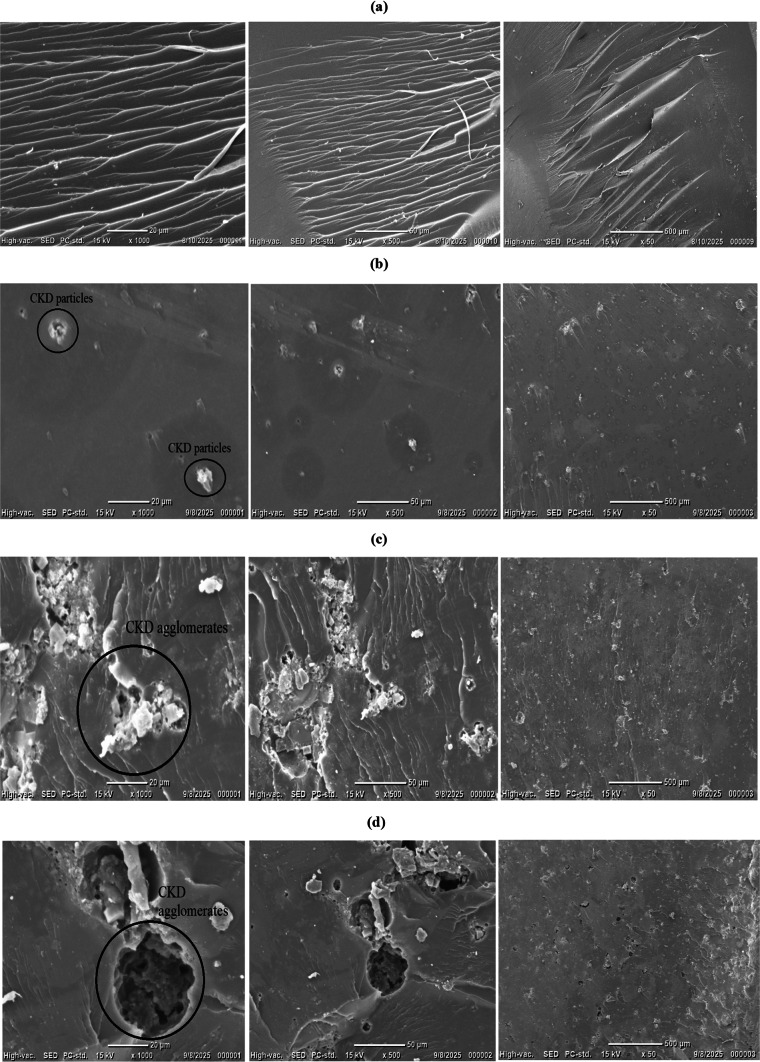
SEM illustrations of (**a**) UP–CKD 0%, (**b**) UP–CKD 2%, (**c**) UP–CKD 6% and (**d**) UP–CKD 10%

As exhibited in the Fig. 6, the neat UP showed a smooth, uniform, and homogenous structure. With the initial insertion of CKD (2%), the defects within the structure start to appear. With rising the CKD (6 and 10%), clustering and agglomeration phenomena occur internally, causing a rougher structure externally. The SEM illustrations were marked, revealing the CKD clusters^18–21,44^. The nature and irregular morphology of native CKD assisted the occurrence of these phenomena, as revealed in previous studies^4,8,10^.

### Mechanical properties interpretation

#### Tensile characteristics

**Fig. 7:**
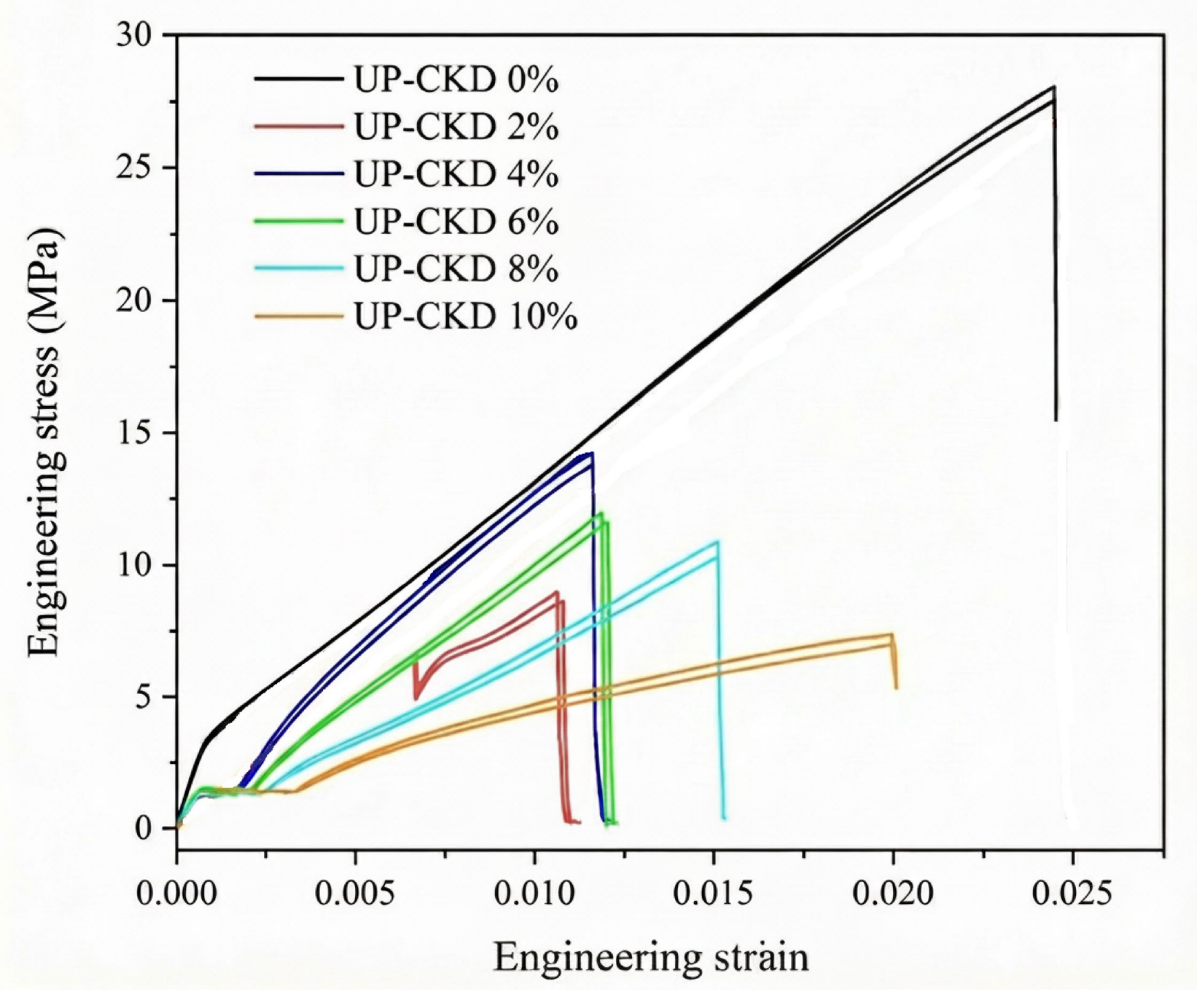
Typical engineering stress–strain curves of the synthesized UP–CKD composites.

As a function of CKD content, both Table 4 and Fig. 7 display the tensile behavior of UP–CKD composites under study. Via dividing the maximum applied force by the area of each sample, the data shows a decrease in the ultimate tensile strength from 27.09 MPa with the first introduction of CKD to the virgin UP-resin matrix to 14.19 MPa ± 2.91 for the maximum content of CKD filler. This value of tensile strength for UP, i.e., ≈ 27 MPa, typically resembles the literature^45^. The data also show a continuous lowering of the tensile strength from 14.19 to 7.31 MPa ± 2.91 with rising the CKD content from 2 to 10%. Concerning the tensile curves, the UP–CKD 2 wt% composite showed a slight deviation from linearity close to the maximum stress, whereas composites containing ≥ 4 wt% CKD failed after an almost linear response. This behavior can be imputed to the transitional role of the 2 wt.% composition, where the UP matrix still dominated the deformation, while the first introduction of CKD particles began to introduce local stress concentrations and limited interfacial damage. Under these low filler loading circumstances, the matrix can undergo some plastic deformation and microcrack blunting before final fracture, which appears macroscopically as a short nonlinear segment^41,46^. In contrast, the progressive increase in CKD filler content from 4 to 10 wt% led to a pronounced reduction in ultimate tensile strength and elongation at break (as will be discussed later), as recorded by Table 4, indicating a more brittle, nearly linear response in which the stiff particulate phase constrains matrix yielding and promotes earlier catastrophic fracture. For discussing the composites’ tensile behavior, the observed decrease in tensile strength with increasing CKD content can be primarily attributed to the introduction of CKD particles that cause increased voids and interfacial gaps within the unsaturated polyester matrix, which reduces wettability and adhesion between the filler and polymer chains^47,48^. This weaker interfacial bonding limits efficient stress transfer and promotes interfacial sliding under tensile load. Additionally, the poor dispersion and agglomeration of CKD particles, as supported by SEM images of the literature^4,8,16^ and the current study (Fig. 6), create localized stress concentration points that facilitate early crack initiation and premature fracture. Also, the irregular geometry of CKD particles^28^ (as can be seen in Fig. 6) impairs their ability to withstand and transfer stress from the polymer matrix, further diminishing composite strength^40^. This comprehensive mechanism aligns with previously reported observations in the earlier literature with rising CKD content^11^. Table 4 also displays Young’s modulus (E). From the table, E values took the same path as the tensile strength, where their values decreased with the first inclusion of CKD and continued to decrease with the enlargement of CKD%. Young’s modulus is a measure of the sample’s rigidity or flexibility when a force is applied lengthwise, and it quantifies the opposition to elastic deformation in the linear region of the stress–strain curve. In the present case, E exhibits a non-monotonic trend where the values were in a fluctuating behavior. E displayed a slight increase of ~ 3.2% at 2% CKD (1154.23 MPa) relative to neat UP (1118.4 MPa), followed by progressive decline up to 76.74% of the rigidity loss at the insertion of 10% CKD (maximum load). The initial enhancement of E at low CKD content (2%) arose from the optimal filler dispersion, allowing favorable particle–matrix contact, which restricted polymer chain mobility and strengthened the matrix via mild stress transfer at the rigid CKD-UP interface. At higher CKD fractions (≥ 4%), excessive filler particles promoted particle–particle interactions and agglomeration, creating stress concentration points, microcracks, and debonding, reducing the effective load-bearing and overall stiffness—mirroring tensile strength reductions (from 27.54 MPa in neat UP to 7.57 MPa at 10% CKD), allowing slipping of the polyester chains by the filler particles^22^. Both the Young’s modulus and tensile strength of the prepared UP–CKD composites behaved like those of cementitious materials doped with unsaturated polyester composites^41^.

**Table 4:** Mechanical properties of UP-CKD composites.

Sample code	Mechanical property					
	Ultimate tensile strength (MPa) ± 2.91	Elongation at break (%) ± 0.28	Young’s modulus (MPa) ± 137	Bending strength (MPa) ± 2.7	Flexural modulus (MPa) ± 0.5	Shore-A hardness ± 0.08
UP–CKD 0%	27.09	2.46	1118.4	25.00	4.68	91.00
UP–CKD 2%	14.19	1.18	1154.23	25.02	4.69	91.30
UP–CKD 4%	13.89	1.22	933.9	22.44	4.20	91.50
UP–CKD 6%	13.31	1.55	718.49	15.00	2.81	91.30
UP-CKD 8%	8.42	2.02	654.51	11.01	2.06	91.10
UP–CKD 10%	7.31	2.91	260.45	11.49	2.15	91.00
F (P)	19.43 (0.010*)	71.93 (1.59 × 10–8*)	2330 (8.94 × 10–10*)	343.7 (2.75 × 10–7*)	787.1 (2.31 × 10–8*)	1.5 (0.314*)

*F* for the ANOVA test.

*p*-value for comparison between the various studied blends.

*Statistically significant at *p* ≤ 0.05.

The elongation% at the break (fracture strain) is the property that indicates the percentage of material’s stretching relative to its original length before it breaks or fractures. Table 4 reveals elongation% values that first decreased from 2.46 to 2.02% ± 0.28 with the rise of the CKD filler in the UP matrix. This decrease may be because of hindering the chains’ movement, where the filler particles act as obstacles/barriers. There was a subsequent increase in the elongation% up to 2.91 with the rise in the filler percent to 10% compared to 2.46 for the pure UP matrix. Nonlinear behavior in the elongation at break (%) of CKD-filled polymer composite was previously also reported^11^. Increasing the cement dust content to 10%, in the as-prepared composite, increased the chance for its particle’s agglomeration until it reached its ultimate peak, which in turn reduced the interaction surface area between it and the matrix besides the possible defects within the sample (like the trapped air bubbles) during its forming facilitating the mobility of the chains increasing the elongation% at the break and responsible for the observed nonlinear behavior. This was supported by the studies in the literature, which have highlighted that inorganic reinforcing fillers with particle sizes exceeding 0.1 μm are prone to degrading and weakening the matrix material due to inadequate interfacial adhesion and the tendency of particles to agglomeration^49^, as in the present case, as shown by Fig. 6.

#### The three-point bending strength

This test determines the ultimate load the sample can withstand before deformation or collapse. Determination of three-point bending (flexural) strength ($\sigma_{B}$) and the flexural modulus ($\sigma_{M}$) reveals a decrease in the ultimate flexural strength values from 25 to 11.49 MPa for pure UP and with rising CKD filler%. The flexural modulus followed the same trend as the flexural strength, where it dropped from 4.68 MPa for raw UP to 2.15 MPa for 10% CKD content (the maximum load) as recorded in Table 4. The present declining behavior is due to the agglomeration and poor dispersion of CKD within the matrix, which results in low adhesion and poor bonding between the CKD filler and the UP matrix^50^. This was promoted by the chemical nature of CKD, as an inert mineral filler, which may not have strong bonding or chemical affinity with the UP matrix itself. This can be linked with the non-uniform surface morphology of untreated CKD. All this progressed the microcracks formation that were developed by the actual presence of voids and internal defects within the matrix. The absorbed bending energy, in this situation, was being deviated and dissipated into various directions, explaining the observed decrease of the flexure stress with the rise of CKD content. The analysis of variance (ANOVA) reported in Table 4 confirmed that the variations in the ultimate tensile strength, elongation (%) at break, Young’s modulus, bending strength, and flexural modulus with CKD content were statistically significant (*p* < 0.05), where they offered P values of 0.011, 1.59 × 10^−8^, 8.94 × 10^−10^, 2.75 × 10^−7^, and 2.31 × 10^−8^, respectively. Considering the same level of confidence of significance, i.e., 5%, the composite under study reveals a high level of statistical significance compared to other reinforced UP–CKD composites^51^.

#### The Shore hardness analysis

**Fig. 8:**
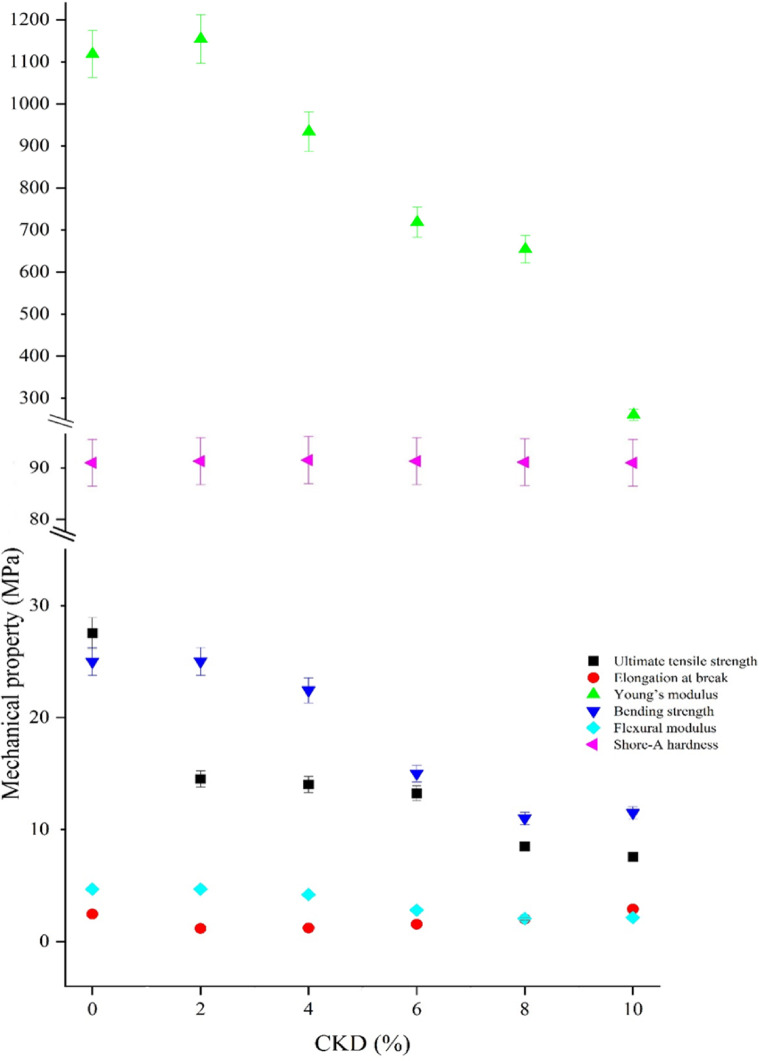
The mechanical properties of the as-prepared UP–CKD composites.

The hardness (shore A) values of the prepared composites are listed in Table 4. The values reveal that the hardness property increased from 91 for the raw UP to 91.50 with the addition of 4% CKD as a filler. After that, the values decreased until returning to 91 again, with the CKD rising to 10%. The first enhancement of the shore hardness was due to the good dispersion of the CKD filler within the structure of the samples, which stiffened their matrix, helping them distribute the applied load and resulting in increased hardness values. Further increase in CKD increased the chances of its agglomeration, which also made the compatibility and interfacial interaction between the UP matrix and CKD filler decrease, showing the observed decline in the hardness values. The hardness followed the inclination to increase after decreasing, as what has been offered by both the flexure strength and flexural modulus. Figure 8 summarizes the mechanical results in Table 4 as a function of increasing the CKD content on the various mechanical characteristics of the UP matrix.

#### Preliminary environmental impact assessment

To quantify the environmental benefits claimed in the study, a precursory cradle-to-gate life cycle assessment (LCA) was carried out following ISO 14,040/44 principles, focusing on global warming potential (GWP) reductions from CKD addition in UP matrix composites. GWP was determined in compliance with Eq. (8)

8 $$GWP_{total} = \sum \left( {m_{i} \times EF_{i} } \right) + E_{proc}$$

where ${m}_{i}$ is the mass fraction of the component $i$ (kg), $E{F}_{i}$ is its emission factor (kg CO₂e/kg from Ecoinvent v3.11), while ${E}_{proc}$ is processing energy emissions (kg CO₂e/kg composite). For a baseline neat UP resin (1 kg), GWP = $1\times 2.5+0.3=2.8$ kg CO₂e (UP: 2.5 kg CO₂e/kg; mixing: 0.3 kg CO₂e/kg Egyptian grid, 0.51 kg CO₂e/kWh). At 10 wt.% CKD substitution (0.9 kg UP + 0.1 kg CKD), GWP = $(0.9\times 2.5)+(0.1\times 0.2)+0.24=2.44$ kg CO₂e, yielding 12.9% reduction; CKD EF = 0.2 kg CO₂e/kg (post-avoided landfill 0.5–1 kg CO₂e/kg minus production credits), cold mixing saves ~ 20% energy (0.24 vs. 0.3 kg CO₂e/kg). Compared to commercial CaCO₃ filler (EF 0.08 kg CO₂e/kg), CKD-UP reveals a 5–8% GWP advantage at 0–10 wt.% due to waste diversion, agreeing with SDGs 11/12 by mitigating Egypt’s ~ 120 tons/day CKD landfilling^52–55^. Biodegradability was excluded as the UP-thermoset matrix (~ 95%) resists degradation, prioritizing filler valorization impacts.

#### The statistical overview and interrelationships between the properties of blend materials

**Fig. 9:**
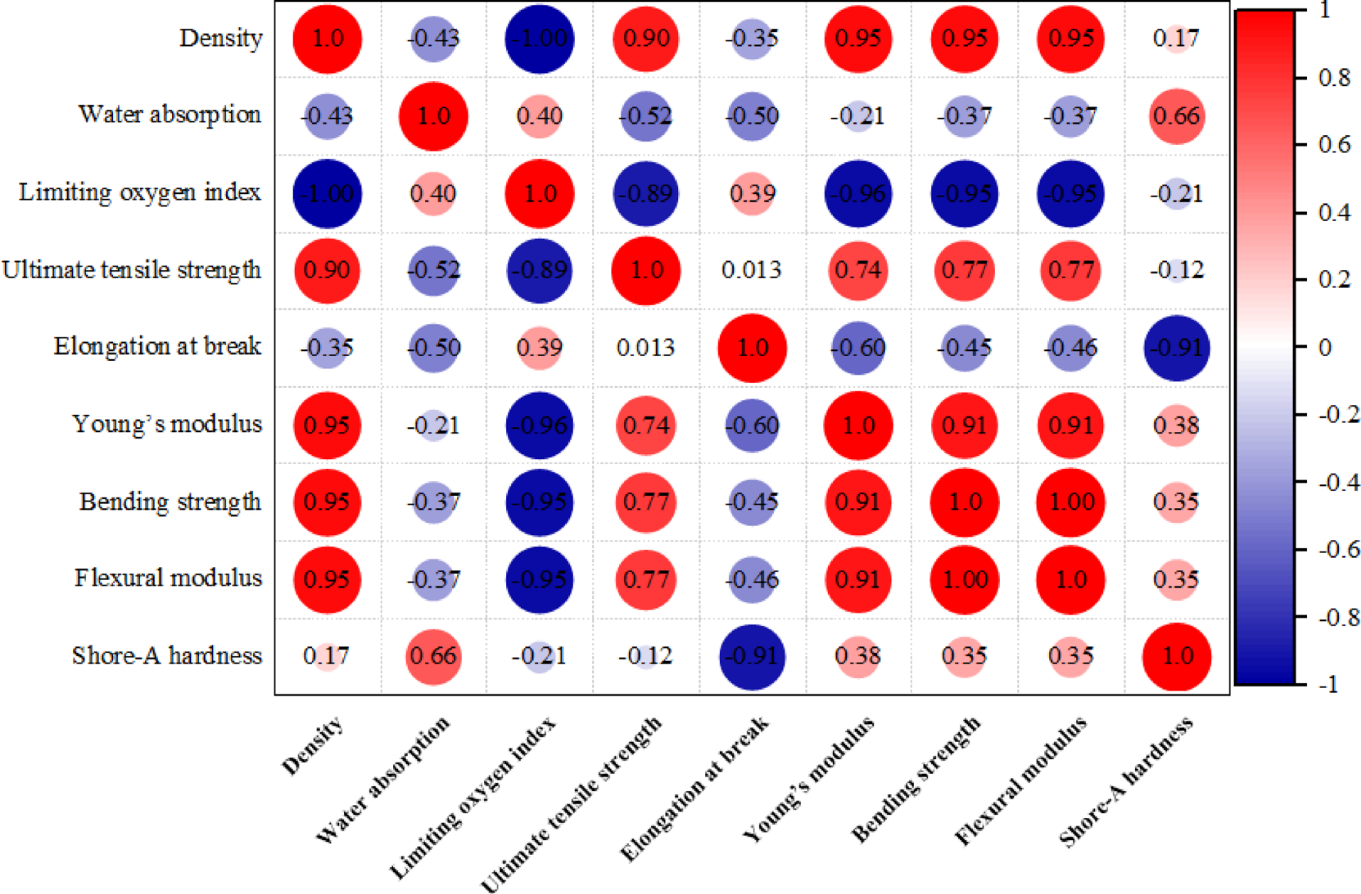
Pearson correlation analysis of varying the CKD ratio with the studied physical and mechanical properties.

Figure 9 exhibits the Pearson correlation matrix plot extending from − 1 (strong negative, blue) to + 1 (strong positive correlation, red), which presents a comprehensive visualization of the interrelationships among the nine tested properties of the as-prepared UP–CKD mixes. The calculated cluster of correlation coefficients delivers rigorous insights and a precise statistical interpretation of how CKD affects UP performance. The density reveals strong positive correlations with the mechanical properties (all ≥ 0.90), suggesting that a decrease in density (due to higher CKD) indicates weaker stiffness and rigidity of the blends. In contrast, a pronounced negative correlation with LOI (− 1.0) suggests high fire resistance (a key parameter in flame retardancy applications). Negative association with water absorption (− 0.43) signifies that lighter blends become more hydrophobic. The LOI reveals an extreme negative correlation with key mechanical characteristics, demonstrating that as the flame retardance improves (higher LOI), the mechanical strength and stiffness tend to decrease because of the reduced matrix continuity and the amended microstructure. The tensile strength is moderately related to other mechanical properties, with no connection to elongation at break (0.013), boosting that strength and ductility in the as-prepared system are largely decoupled and behave independently. The negative correlation between elongation and other mechanical properties (≤ − 0.60) emphasizes the inherent challenge of balancing the toughness and ductility of the prepared blends when CKD is incorporated. The hardness positively correlates with other computed strength properties (Young’s and flexural moduli), recommending that surface attributes are improved without comprehensive improvements in bulk toughness. Finally and compared to the literature, the synthesized UP–CKD composite can find applications in both architecture and chemical fields^56,57^. Souza et al. used a composite that typically resembles the as-prepared one in applications like benches and tables manufacturing, where there is no need for significant mechanical characteristics in these structures, which saves costs and provides durability^35^.

## Conclusion

The properties of micro-sized non-fibrous CKD filler, in the unsaturated (UP) thermoset matrix, were studied and evaluated. This research established that untreated CKD can be successfully incorporated into UP composites via cold mixing without requiring solvents or external heating—a processing pathway previously unexplored in CKD-polymer composite literature. Both XRD and IR spectra analyses of the as-prepared composites showed the existence of distinct peaks of UP and CKD, which reflect their physical interaction. The reduction of the experimental density from 2 to 1.08 g/cm^3^ with further addition of CKD filler allowed the possibility of using the synthesized composite in light engineering materials that require low weight. Although the degradation temperature decreased with the inclusion of CKD up to 10%, the composite was thermally stable up to 250 °C, allowing the use of the UP–CKD composite in thermally stable applications within this temperature range. The water absorption results revealed that the prepared UP–CKD composite did not absorb water more than 0.5% (as a maximum), revealing the stability and low water uptake of the prepared composite. Testing the flammability of the prepared composites showed an improvement in the UP-fire resistance properties with the increase in CKD% due to the increase in its oxygen demand for fire (LOI) above the oxygen percentage (21%) in the air. The studied mechanical properties in terms of ultimate tensile strength, Young’s modulus, bending (flexural) strength, and flexural modulus decreased from 27.54 to 7.57 MPa, from 1118.4 to 260.45 MPa, from 25.00 to 11.49 MPa, and from 4.68 to 2.15 MPa, respectively, with increasing CKD. Although general mechanical properties systematically declined with CKD loading, due to poor interfacial adhesion and agglomeration (revealed by SEM illustrations), low loadings (e.g., 2 wt% CKD) offered an optimal balance with ~ 3.2% Young’s modulus enhancement from improved dispersion, making it suitable for lightweight, semi-rigid applications. Higher fractions of CKD (10 wt%) prioritized fire resistance (LOI > 21%) over mechanics, as confirmed by statistically significant trends (ANOVA, *p* < 0.05). This counter-intuitive relationship between enhanced fire resistance and mechanical loss, combined with the low-emission processing methodology, distinguishes this work from prior CKD-composite investigations and provides important guidance for industrial applications o’f CKD waste streams.

## Data Availability

All data generated or analyzed during this study are included in this published article.
